# The blur horopter: Retinal conjugate surface in binocular viewing

**DOI:** 10.1167/jov.21.3.8

**Published:** 2021-03-04

**Authors:** Agostino Gibaldi, Vivek Labhishetty, Larry N. Thibos, Martin S. Banks

**Affiliations:** 1 School of Optometry, University of California at Berkeley, Berkeley, CA, USA; 2 School of Optometry, University of California at Berkeley, Berkeley, CA, USA; 3 School of Optometry, Indiana University, Bloomington, IN, USA, USA; 4 School of Optometry, Vision Science Program, University of California at Berkeley, Berkeley, CA, USA

**Keywords:** retinal conjugate surface, wavefront aberrations, parafoveal and peripheral retina, accommodation, point-spread function, binocular horopter, natural-scene statistics

## Abstract

From measurements of wavefront aberrations in 16 emmetropic eyes, we calculated where objects in the world create best-focused images across the central 27${}^{\circ }$ (diameter) of the retina. This is the retinal conjugate surface. We calculated how the surface changes as the eye accommodates from near to far and found that it mostly maintains its shape. The conjugate surface is pitched top-back, meaning that the upper visual field is relatively hyperopic compared to the lower field. We extended the measurements of best image quality into the binocular domain by considering how the retinal conjugate surfaces for the two eyes overlap in binocular viewing. We call this binocular extension the *blur horopter*. We show that in combining the two images with possibly different sharpness, the visual system creates a larger depth of field of apparently sharp images than occurs with monocular viewing. We examined similarities between the blur horopter and its analog in binocular vision: the binocular horopter. We compared these horopters to the statistics of the natural visual environment. The binocular horopter and scene statistics are strikingly similar. The blur horopter and natural statistics are qualitatively, but not quantitatively, similar. Finally, we used the measurements to refine what is commonly referred to as the zone of clear single binocular vision.

## Introduction

When humans fixate an object, they adjust the orientations of the two eyes so that the lines of sight intersect at the object and they adjust focus to ensure a sharp retinal image. With accurate alignment (vergence) and focus (accommodation), clear single binocular is established. Without such alignment and focus, images may appear double, blurry, or both. These issues and their solution are captured by the *zone of clear single binocular vision* (Hofstetter, 1945). In this article, we concentrate on best image quality across the central visual field and how images of possibly different sharpness are combined binocularly.

**Figure 1.**
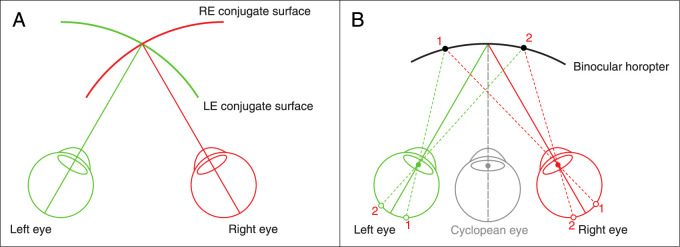
Retinal conjugate surfaces and the binocular horopter. (**A**) Retinal conjugate surfaces. Green and red are theoretical conjugate surfaces for the left and right eyes, respectively. They represent the object positions that create best-focused images across the retinas. (**B**) Binocular horopter. While the eyes fixate centrally, Objects 1 and 2 create retinal Images 1 and 2. If the image points in the two eyes are corresponding points, Objects 1 and 2 lie on the binocular horopter. The fused binocular percept is referenced to the cyclopean eye.

Image formation in the eye is subject to a variety of optical imperfections, including diffraction, defocus, astigmatism, higher-order monochromatic aberrations, and chromatic aberration. Despite these imperfections, there is a surface in the world that creates the sharpest image across the retina given the eye's current accommodative state and pupil size. This surface is the optical conjugate of the retinal surface. It is the *retinal conjugate surface*. There is one for each eye, as shown in Figure 1A.

In binocular vision, there are pairs of points in the two eyes that are retinally corresponding points. Stimulation of these pairs yields perception of identical visual directions from the two eyes. The external manifestation of those points is the *binocular horopter*: the positions in space where rays from a corresponding pair intersect (Figure 1B). When objects are on or near the horopter, single, fused vision is guaranteed (Fischer, 1924; Ogle, 1950) and stereoscopic depth perception is most precise (Ogle, 1953; Blakemore, 1970; Schumer & Julesz, 1984). Thus, the binocular horopter is the region in space for which binocular vision is best.

Binocular disparity and blur at the retinas are subject to essentially the same viewing geometry. Disparity arises from differences in the left- and right-eye viewpoints while blur arises from differences in viewpoints across the pupil (Schechner & Kiryati, 2000; Held, Cooper, O'Brien, & Banks, 2010). Said another way, disparity and blur are both based on triangulation. Inspired by this underlying similarity, we investigate the relationship between the binocular horopter (where binocular vision is best) and the retinal conjugate surface (where image quality is best). We first determine the retinal conjugate surface in the world for several accommodation distances. We examine whether the conjugate surface conforms to the statistics of the natural visual environment. We next consider how images of perhaps different quality are combined binocularly. In this, we introduce the *blur horopter*; it is the surface in the world where objects appear sharpest in binocular viewing. We show that it is a thick surface bounded in near and far viewing by different parts of the two retinal conjugate surfaces. We then examine the relationship between the blur and binocular horopters. Finally, we refine the concept of the zone of clear single binocular vision to give it a more quantitative basis.

## Methods

Measurements of wavefront aberrations were obtained with the *Indiana Scanning Aberrometer for Wavefronts* (I SAW) (Liu, Sreenivasan, & Thibos, 2016; Liu & Thibos, 2017). Subjects were 16 emmetropes (19–36 years old; mean spherical equivalent refraction = +0.2 diopters [D], *SD* = 0.3 D). Data are from the left eyes across the central 27${}^{\circ }$ (diameter) of the visual field. Liu and Thibos obtained aberration data from each subject at 37 retinal locations, including the fovea. A high-contrast letter E was used as the fixation and accommodative stimulus. During the measurements, subjects performed a Tumbling-E acuity task, and this ensured accurate fixation and accommodation. Liu and Thibos also obtained data for eight accommodative stimulus distances (−1 to +6 D in 1-D steps). Pupil size was measured continuously. A detailed description of the instrument and procedure for collecting the data is presented by Liu et al. (2016). It is important to note that we use diopters as the measure of distance throughout the current article.

## Best-focus distance

We sought to determine the surface in the world that creates the best-focused image across the retina. In this section, we explain how we determined that surface and how its shape and distance depend on the accommodative stimulus.

The human eye is subject to significant optical imperfections, including diffraction, defocus, astigmatism, and a host of higher-order aberrations, so we need to take them into account when determining “best focus.” An important consideration is astigmatism. Image formation for objects that are not on the optical axis of a simple lens exhibits *astigmatism of oblique incidence* (Atchison & Smith, 2000; Liu & Thibos, 2016). To describe this effect, it is useful to consider rays propagating from an object point toward the retina in two special planes. The first is the *tangential plane*, the plane containing the object point, the chief ray (the ray passing from the object through the eye's nodal point), and the optical axis of the lens. Light rays propagating in this plane are tangential rays. The second is the *sagittal plane*, which is orthogonal to the tangential plane and also contains the chief ray. Sagittal rays propagate in this plane, but we prefer to call them radial rays because, in analyses described later in the article, we consider images formed by tangential and radial lines in the world. Tangential and radial rays form best focus at different distances along the chief ray. Specifically, tangential lines in the world (i.e., lines perpendicular to radii from the fovea) are focused more myopically than radial lines. The differing best-focus positions produce astigmatism. The positions of best focus are typically on curved surfaces behind the lens creating *field curvature*, with greater curvature for tangential rays.

**Figure 2.**
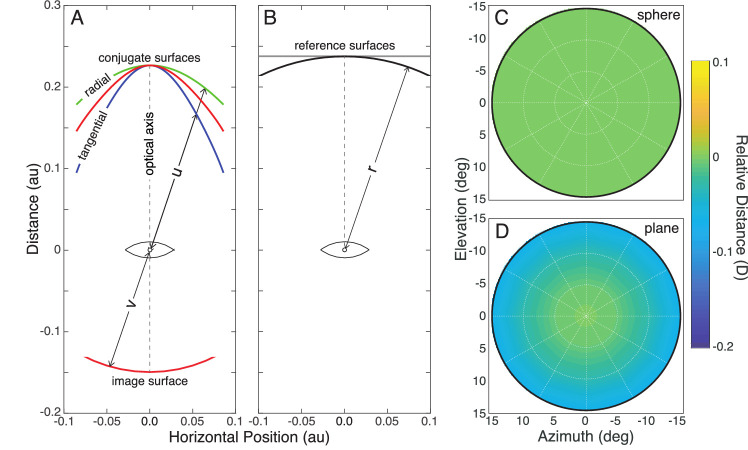
Conjugate and reference surfaces. (**A**) Retinal conjugate surfaces for an optical system with oblique-incidence astigmatism. The image surface is a sphere centered at the optical center of the lens. The green curve corresponds to the conjugate surface for radial lines; the blue curve corresponds to the surface for tangential lines. The difference is astigmatism. The area between the two conjugate surfaces is the interval of Sturm. The red curve is the circle of least confusion where focus for tangential and radial lines is collectively best. u and v are the distances from the lens center to the image surface and conjugate surface, respectively. (**B**) Spherical and planar conjugate surfaces. A sphere with radius r (black), centered on the eye's entrance pupil, has constant distance. We use it throughout as the reference surface for computing object distance at different eccentricities. When the conjugate surface is identical to the reference surface, the relative object distance is zero everywhere. A hypothetical planar conjugate surface (gray) is also shown. (**C**) Heatmap, centered on the fovea. (**D**) Heatmap showing how relative distance varies across the field if the conjugate surface is a plane and the reference surface is the sphere. Relative distance is now non-zero away from the fovea.

Figure 2 shows how relative object distance (object distance at fovea set to 0 D) varies across the visual field when the conjugate and reference surfaces are both spheres of radius r. Relative distance is zero everywhere.

Figure 2A shows retinal conjugate surfaces for a thin lens and a spherical image surface. The green curve is where radial lines are brought to best focus, and the blue curve is where tangential lines are. The difference between the two curves is due to oblique-incidence astigmatism. The red curve is the circle of least confusion where the two foci are in relatively best focus. Notice that the surfaces exhibit field curvature.

Not surprisingly, human eyes exhibit significant oblique-incidence astigmatism (Ferree, Rand, & Hardy, 1931; Rempt, Hoogerheide, & Hoogenboom, 1971; Gustafsson, Terenius, Buchheister, & Unsbo, 2001; Jaeken & Artal, 2012; Liu & Thibos, 2017). For example, Gustafsson et al. (2001) reported that emmetropic subjects have 7–10 D of astigmatism at $\pm 60^{\circ }$ from the fovea along the horizontal meridian. The axis of the oblique-incidence astigmatism is radial, and the magnitude increases roughly in proportion to the square of eccentricity (Liu & Thibos, 2017). This aberration has a substantial effect on image quality in the periphery, so it is important to take it into account when estimating the best focal distance in various field locations.

Figure 2B shows that we quantify distance radially along rays emanating from the eye's entrance pupil. In our calculations, we used point-spread functions (PSFs) to incorporate all monochromatic aberrations and used line-spread functions (LSFs) to emphasize the consequences of oblique-incidence astigmatism.

**Figure 3.**
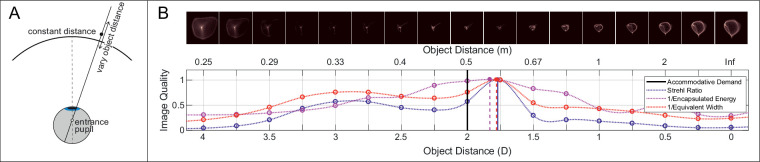
Search for best focus. (**A**) Vary object distance to find distance of best focus. For each retinal location, the virtual object is moved in depth along the ray from that location through the eye's nodal point. Distance is measured radially, so objects on the spherical surface shown would have constant distance. (**B**) Best-focus distance using different image-quality metrics. For each field location and accommodative stimulus, we computed the point-spread function (PSF) as a function of object distance. The top panels show the PSFs for one subject for each object distance for an accommodative stimulus of +2 D and a location 5${}^{\circ }$ nasal from the fovea. For visualization purposes, each PSF is normalized by its maximum value. The results (normalized to 1) are shown in the lower panel for Strehl Ratio (blue), Encircled Energy (purple), and Equivalent Width (red). From the center of mass of the resulting curve, we found the distance providing the highest quality (vertical lines) for each metric.

We used Zernike coefficients from the I SAW data to reconstruct the wavefront for every field position, accommodative stimulus, and subject. The coefficients included all aberrations from 2nd to 40th order. To find the best-focus distance for each wavefront, we changed the second-order coefficient of defocus ($c_{2}^{0}$; Mahajan, 1994) in 0.25-D steps, applying each step to the measured aberration map (Tarrant, Roorda, & Wildsoet, 2010). This was done by converting from microns (μ) to diopters (D):

(1) $$c_{2}^{0}\left(D\right)=\frac{4\sqrt{3}c_{2}^{0}\left(\mu \right)}{r^{2}}$$

where r is the pupil radius in millimeters. The complex pupil function was calculated from the pupil size measured by I SAW. The PSF was then computed as the squared magnitude of the Fourier transform of the complex pupil function (Figure 3B). Radial and tangential LSFs were computed by convolving the appropriate PSF with radial or tangential lines and acquiring the cross sections.

The retinal image formed by an object point (the PSF) is complicated, so it not clear what aspect of the PSF constitutes “best focus.” To solve this problem, several metrics have been proposed to quantify retinal-image quality (Cheng, Bradley, & Thibos, 2004; Thibos, Hong, Bradley, & Applegate, 2004). Using these metrics, one can adjust the distance of an object point until the quality of the corresponding retinal image is maximized (Tarrant et al., 2010). That distance constitutes a point on the best-focused surface in the world. To do this, we employed three common metrics: *Strehl Ratio*, *Encircled Energy*, and *Equivalent Width*.

The Strehl Ratio (SR) is the ratio of the peak value of the observed PSF to the peak value of the PSF of the diffraction-limited (i.e., unaberrated) eye with the same pupil diameter:

(2) $$SR=\frac{peak(PSF_{o}\left(x,y\right))}{peak(PSF_{d}\left(x,y\right))}$$

where $peak(PSF_{o})$ and $peak(PSF_{d})$ are respectively the peak values of the observed and diffraction-limited PSFs. Strehl Ratios approaching 1 indicate high image quality. The Visual Strehl Ratio is based on the Strehl Ratio but also takes into account the neural transfer function. It is a good predictor of perceived image quality (Cheng et al., 2004; Thibos et al., 2004), but we unfortunately could not use it because the neural transfer functions for off-axis field positions are not known with sufficient accuracy.

Encircled Energy (EE) is the radius of the circle centered on the PSF peak that includes 50% of the intensity in the observed PSF:

(3) $$\begin{array}{c} \;\;\;\;\;\;\;\;\;\;\;\;\;EE=\underset{r}{\mathrm{argmin}}\left\{\int_{0}^{2\pi }\int_{0}^{r}\left[PSF_{N}\left(x_{0},y_{0}\right)-0.5\right]drd\theta \right\}\;\; \end{array}$$

where $x_{0}$ and $y_{0}$ are the coordinates of the peak and $PSF_{N}$ is the PSF normalized to unit volume. Smaller diameters correspond to better image quality.

Equivalent Width (EW) is the radius of the cylinder that has the same volume and height as the observed PSF:

(4) $$EW=\frac{1}{\sqrt{\pi PSF_{N}\left(x_{0},y_{0}\right)}}$$

Again, smaller widths correspond to better image quality.

We used those three image-quality metrics to find the object distance that maximized image quality for every retinal position and accommodative stimulus. The approach is illustrated in Figure 3. We projected rays from a given field position through the eye's nodal point onto the retina. We then varied object distance along such rays in 0.25-D steps over a range of ±2 D (centered at the distance of the accommodative stimulus). Because of the eye's aberrations, rays projected through the pupil onto the retina will not intersect at a point; instead, light is distributed as the PSF. Thus, our search is to find the distance that would generate best image quality for the given retinal position. We repeat this for every retinal position to find the set of distances generating best quality. The upper panel of Figure 3B shows the PSFs associated with each distance for one field location (5${}^{\circ }$ nasal) and accommodative stimulus (+2 D). The lower panel shows how the three metrics vary smoothly with changes in distance reaching a maximum in this case at ∼+1.75 D. The difference between the accommodative stimulus (2D) and the distance of best image quality is an accommodative lag (0.25 D); a difference in the opposite direction would be an accommodative lead (Morgan, 1944; Charman, 1982).

**Figure 4.**
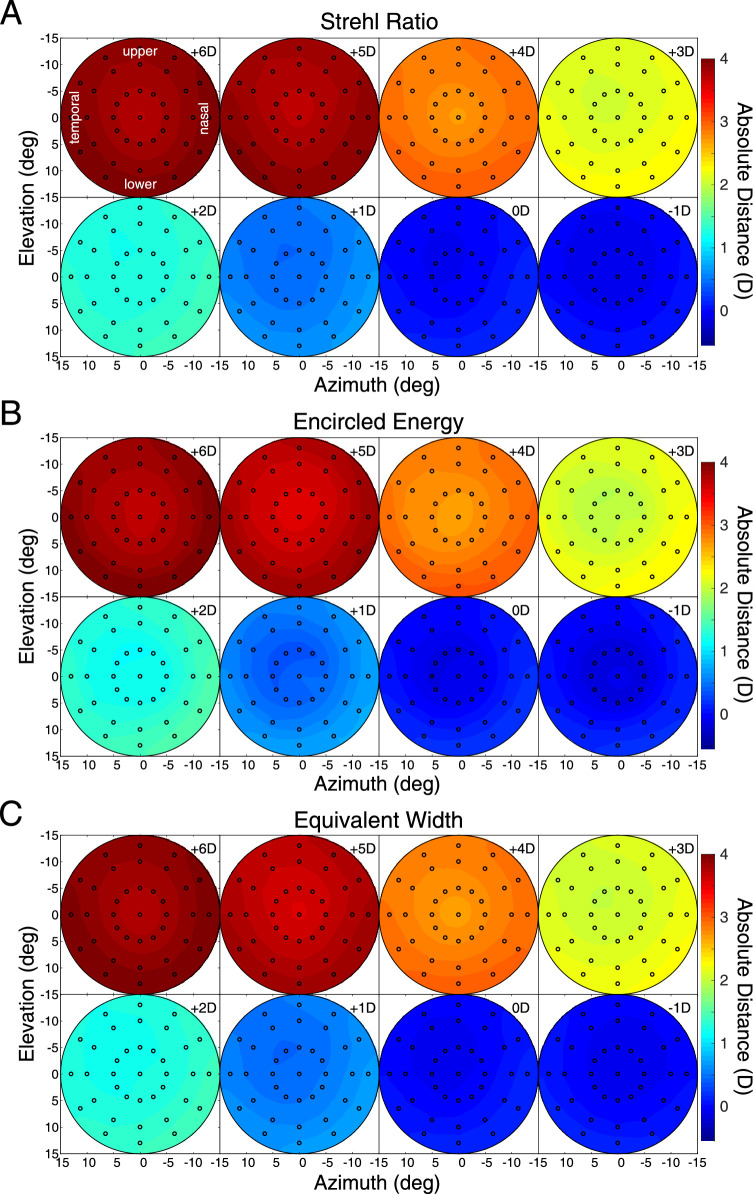
Best absolute distance. The data are medians across subjects. The heatmaps show the object distance corresponding to best image quality for every field point. (**A**) Results when the image-quality metric is the Strehl Ratio. Different panels show the data for accommodative stimuli of −1 to +6 D. The field positions for all the panels (and all subsequent heatmaps) are indicated in the upper left panel. (**B**) Results with Encircled Energy. (**C**) Results with Equivalent Width. One subject had an apparently erroneous measurement for the −1D stimulus so that person's data are not in the −1D panels.

**Figure 5.**
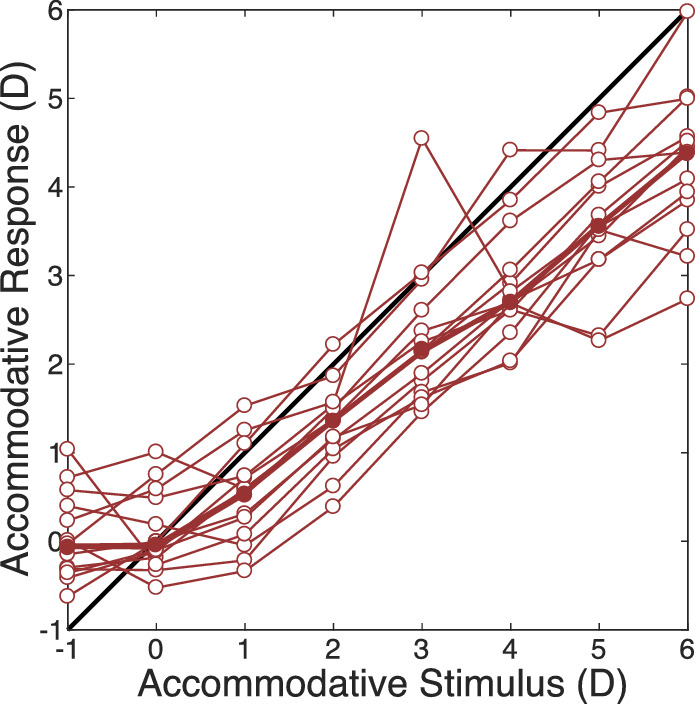
Accommodative stimulus-response curves. The absolute distance that maximized the Strehl Ratio at the fovea is plotted as a function of stimulus distance. The black line is where response equals stimulus. The thin lines and unfilled symbols represent the data from individual subjects. The thick lines and filled symbols are the medians at each distance. One subject had an apparently erroneous measurement for the −1-D stimulus so that person's data are not in the −1-D panel.

All the heatmaps in the current article are centered on the fovea. The results of this analysis, averaged across the subjects, are shown in Figure 4. The upper, middle, and lower parts of the figure show the object distances that maximize Strehl Ratio, Encircled Energy, and Equivalent Width, respectively. The individual panels show the results for the eight accommodative stimuli. A couple of things stand out. First, the three metrics yield substantially similar results. This is comforting because it means that conclusions will not depend on the image-quality metric one chooses. Second, lags and leads of accommodation are evident (Morgan, 1944; Charman, 1982): lags where the eye (specifically, the fovea) appears to be focused farther than the accommodative stimulus and leads where it appears to be focused nearer than the stimulus. Figure 5 shows the lags and leads as a function of stimulus distance. The thin lines and open symbols are the absolute distances at the fovea that maximized the Strehl Ratio for each subject. The thick lines and filled symbols are the medians at each distance. The lag is ∼1.5 D when the stimulus is +6 D and the lead is ∼1 D when the stimulus is −1 D. Those are consistent with previous reports (Liu et al., 2016; Plainis, Ginis, & Pallikaris, 2005).

**Figure 6.**
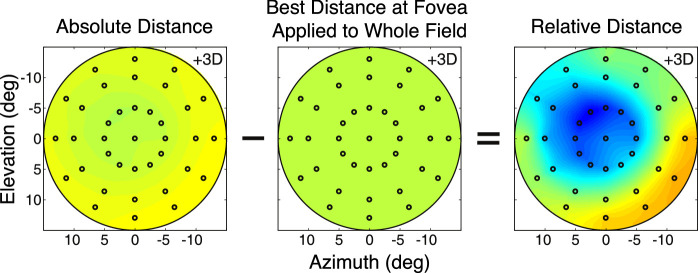
Computing relative distance. Best absolute distance is obtained for each subject and accommodative stimulus using the Strehl Ratio (left). Best distance at the fovea (middle) is then subtracted from best distance at every other field location to yield a relative distance map (right). The relative-distance map reveals changes in best-focused distance across the visual field. The data here are from one subject when the accommodative stimulus was +3 D. The heatmaps for the left and middle panels use a larger range than the map for the right panel. Positive azimuths are temporal (left) points in the visual field and negative values are nasal (right). Positive elevations are inferior (lower) points in the visual field and negative are superior (upper).

**Figure 7.**
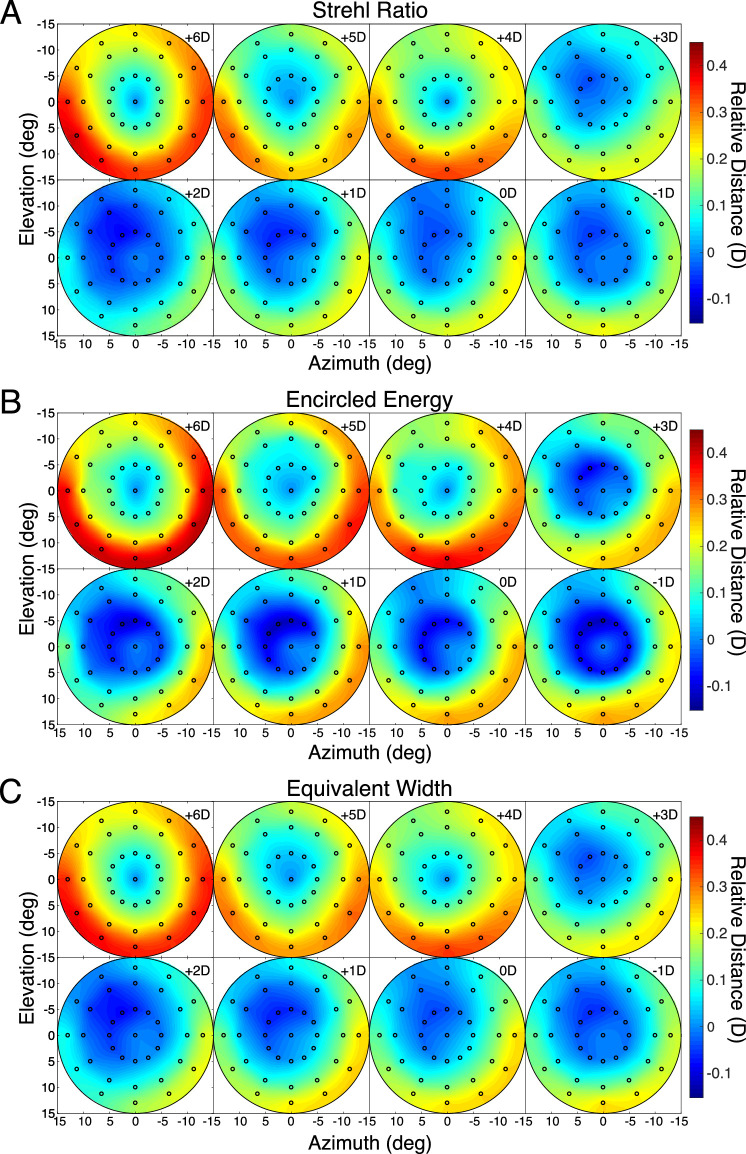
Best relative distance. The data are medians across subjects. The heatmaps show the best distance in diopters relative to the distance at which the fovea yields best image quality. (**A**) Results when the image-quality metric is the Strehl Ratio. Different panels show the data for accommodative stimuli of −1 to +6 D. (**B**) Results with Encircled Energy. (**C**) Results with Equivalent Width. Positive values of relative distance are nearer than negative values.

We next subtracted the lag-lead errors at the fovea from all measured points. We did this for two reasons. First, the lags and leads are relatively large errors, so they obscure variations in best-focus distance across the visual field. By subtracting them, we can see variations across the field at a finer scale. Second, in a forthcoming study, we report that the lags and leads are significantly larger when measured with objective methods like I SAW than lags and leads measured with subjective methods (i.e., best-perceived image quality) (Labhishetty, Cholewiak, & Banks, 2020). This means that accommodative lags and leads are much smaller when assessed in terms of visual performance than when assessed from retinal reflection techniques such as I SAW. Accordingly, we subtracted the absolute best-focus distance at the fovea from the absolute distances observed at the other field positions (Figure 6). We did this subject by subject. The results—the relative distances for best image quality across the visual field—are shown in Figure 7. There are a few noteworthy points. First, the three metrics again yielded quite similar results, which means that conclusions will not depend on the choice of image-quality metric. Second, the pattern of best relative distances across the visual field was quite similar from one accommodative stimulus distance to another. This supports the conclusion by Liu et al. (2016) that best focus across the visual field is similar whether the eye accommodates far or near. Third, there was a consistent trend for best relative distance to be nearer in the lower than in the upper field and to be slightly nearer in the right field than in the left field. Said another way, the emmetropic eye is relatively myopically focused in the lower and nasal fields relative to the upper and temporal fields.

We also examined what happens when one combines data across subjects according to the accommodative responses rather than the accommodative stimuli. The results were quite similar to those in Figure 7 except for a roughly 1-D shift from one panel to the next. That is to say, the data for a +3D response looked very similar to the data for a +4D stimulus.

**Figure 8.**
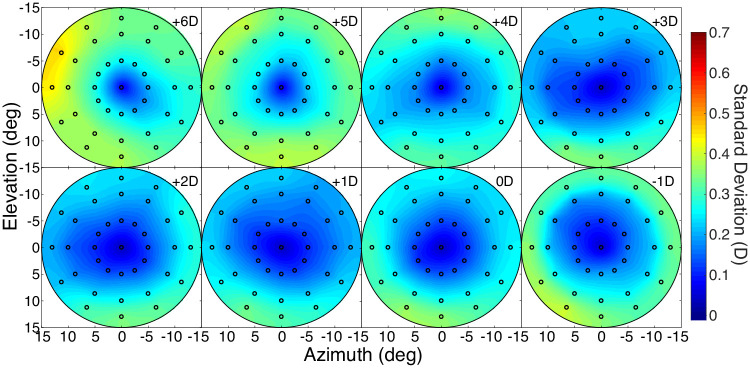
Variation of relative best-focus distance across subjects for each accommodative stimulus distance. The absolute best-focus distance at the fovea was subtracted for each emmetropic subject at each accommodative stimulus, and then the standard deviation was computed from the set of relative distances at each position in the visual field.

We examined the variation in best image quality across subjects. Figure 8 shows standard deviations of relative distance across subjects for each accommodative stimulus distance. To compute the standard deviations, we again first subtracted the best-focused distance at the fovea subject by subject as in Figure 7 and then calculated standard deviations at each field location from those values. The variation in relative distance increased with greater eccentricity but was generally small, ranging from ∼0 to 0.4 D. This means that best relative distance does not vary much from one emmetropic subject to another.

**Figure 9.**
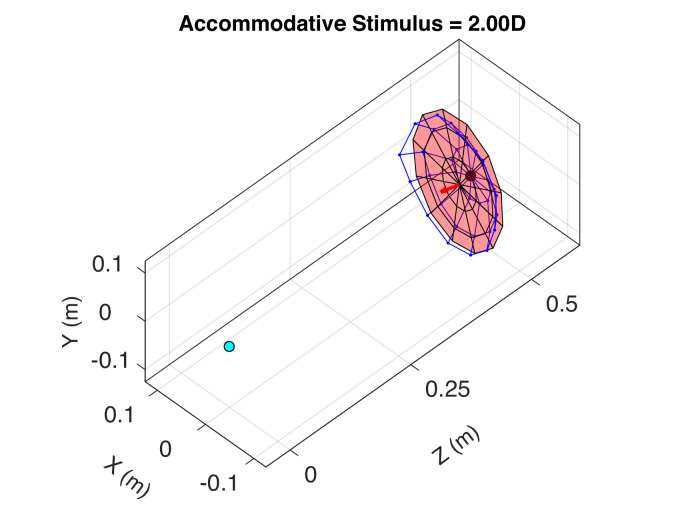
Retinal conjugate surface and accommodation. This is an animation of the best-focus surface averaged across subjects for accommodative stimuli of +2 to +4 D. In the computation, we used PSFs and the Strehl Ratio. The blue circle represents the left eye and the red circle the accommodative stimulus (corrected for lag and lead errors at the fovea). The blue mesh is the best-focus surface. The pink plane is the plane that best fits the mesh. The arrow is that plane's surface normal. Upper visual field is on the top and nasal field on the right. Please visit the article on the journal website to play the movie.

**Figure 10.**
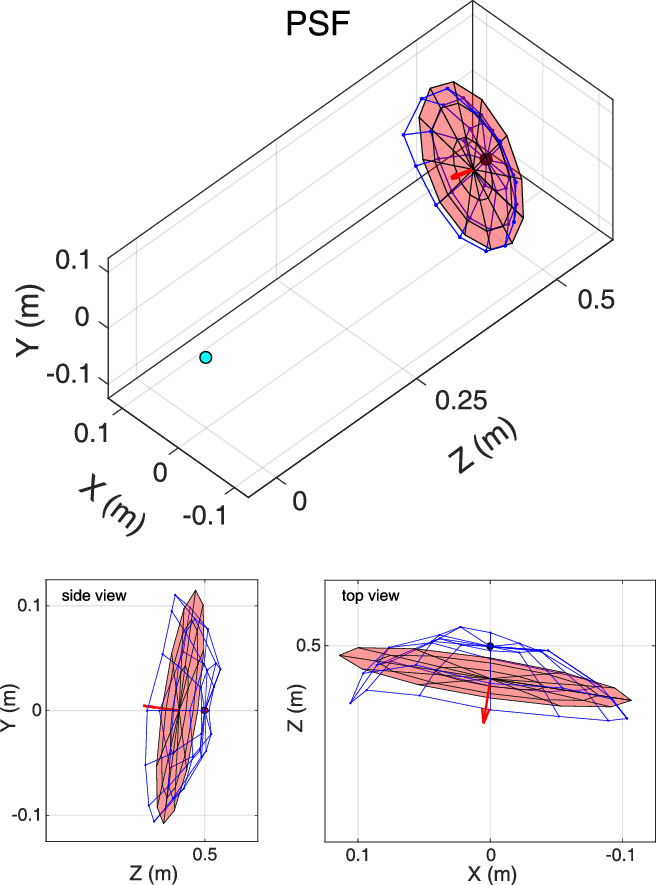
Retinal conjugate surface for an accommodative stimulus of +2 D. Clockwise from above the three panels are viewpoints that are behind and to the right of the eye, above the eye, and to the right of the eye. In the computation, we again used PSFs and the Strehl Ratio. The blue mesh is the best-focus surface (medians across subjects), and the pink plane is the plane that best fits the mesh. The red arrow is the surface normal of that plane. Field positions for the left eye are indicated.

We converted the distances associated with best focus into three-dimensional surfaces (*XYZ* with the origin at the eye's nodal point). We did this for every accommodative stimulus and subject. Linear interpolation along each ray pinpointed best distance from the discrete sampling. The 0 and −1-D stimulus distances were excluded because the associated surfaces of best image quality would have had points more distant than infinity. Figure 9 is an animation showing the retinal conjugate surface, averaged across subjects, for accommodative stimuli of +2 to +4 D. The eye is represented by the blue dot and the accommodative stimulus by the red dot. The best surface, represented by the blue mesh, is derived directly from the best relative distances shown in Figure 7. A plane fit to the surface (using a Levenberg-Marquardt algorithm; Moré, 1978) is shown in pink along with its surface normal. Note that the conjugate surface largely maintains its shape across the 2-D range of accommodative stimuli and that it is pitched top-back and slanted temporal back (see Figure 10).

Figure 10 is a similar representation of the retinal conjugate surface. The stimulus distance is +2 D. Clockwise from the top, the figure shows the view from above and to the right of the eye, from above the eye, and from the right. Again, the blue mesh represents the median best distances across the emmetropic subjects. The pink surface is the best-fitting plane. It is pitched top-back and rotated temporal back. The top-back pitch is consistent with previous measurements of refraction in the upper and lower fields (Ehsaei, Mallen, Chisholm, & Pacey, 2011; Seidemann, Schaeffel, Guirao, Lopez-Gil, & Artal, 2002). This surface represents the positions in the three-dimensional space that yield the highest image quality when the distance to the accommodative stimulus is 50 cm (+2 D). Obviously, the surface moves closer to the eye when the stimulus is closer and farther from the eye when the stimulus is farther. But it mostly retains its shape: somewhat concave, pitched top-back, and rotated nasal side back. To determine if the top-back pitch is statistically reliable, we compared the distances of best focus for the nine upper-field positions with those for the nine lower-field positions and found that the upper ones were significantly farther than the lower (*t* test, $p<10^{-10}$, df=1,294). To determine if the temporal-back rotation is reliable, we compared the distances for the nine temporal field positions with those of the nine nasal field positions and found that the temporal ones were significantly farther ($p<10^{-8}$, df=1,294).

## Astigmatism

As noted earlier, image formation in the periphery is subject to significant astigmatism. The magnitude of oblique-incidence astigmatism is expected to be roughly proportional to the square of the eccentricity, a relation that is confirmed by empirical measurements (Ferree et al., 1931; Liu & Thibos, 2017).

**Figure 11.**
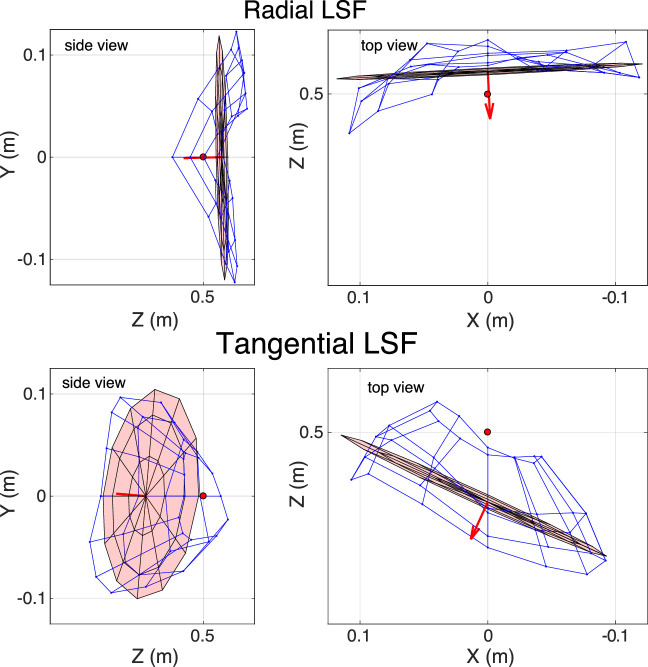
Surface of best-focus distance computed with radial and tangential LSFs. Accommodative stimulus is +2 D. Data averaged across subjects. The upper and lower halves show the results with radial and tangential LSFs, respectively. The left panels are views from the right of the eye. The right panels are views from above. Blue meshes represent the best-focus surfaces. Pink planes are fits to those surfaces. The red circle represents the accommodative stimulus.

The axis of oblique-incidence astigmatism is tangential (i.e., vertical to the left and right of the fovea and horizontal above and below). So from this optical effect alone, we expect tangential lines in the world to be more myopically focused than radial lines. We tested this expectation by calculating the retinal conjugate surfaces for tangential and radial lines. To do this, we derived tangential and radial LSFs for each subject, field position, and accommodative stimulus and then found the object distances that yielded the largest Strehl Ratios (the peak of the observed LSF divided by the peak of the diffraction-limited LSF). As expected, radial LSFs yielded a conjugate surface that is less myopically focused than tangential LSFs (Figure 11), so the radial surface is much less concave than the tangential surface. It is interesting to note that the best distances for the tangential and radial LSFs are more similar in the temporal visual field than in the nasal field. This is in part caused by the deviation between the eye's optical axis (the line perpendicular to the cornea that intersects the center of the entrance pupil) and the visual axis (the line from the fixated point in the world to the fovea). The optical axis in most adult humans deviates $∼5^{\circ }$ temporally from the visual axis (Artal, 2014). As a consequence, the midpoint for oblique-incidence astigmatism is closer to the optical axis than to the visual axis (Liu & Thibos, 2017). Our astigmatism data are consistent with this asymmetry relative to the visual axis.

## Image quality

**Figure 12.**
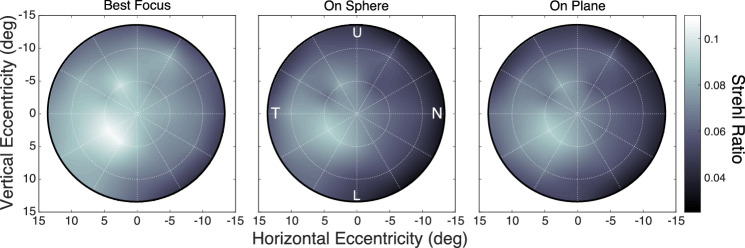
Image quality across the visual field. Each panel shows the Strehl Ratio for all field positions, for an accommodative stimulus of 2 D, averaged across subjects. Lighter values correspond to higher ratios and therefore better image quality. From left to right, the panels show the Strehl ratios at best-focus distance, the ratios relative to a spherical surface (with the fovea in best focus), and the ratios relative to a planar surface (again with the fovea in best focus). The spherical and planar reference surfaces are the same as those in Figure 2C and D. U, N, L, and T indicate upper, nasal, lower, and temporal visual field, respectively.

As we have seen, some aberrations—especially astigmatism—are greater in the peripheral field than in the central field. We also observed that the consequences of these aberrations do not change substantially with changes in accommodative state (Figure 7). We next estimated image quality across the visual field by computing the maximum of the Strehl Ratio at each field location. The left panel of Figure 12 shows the results. The maximum ratio for each field location is shown, averaged across accommodative stimuli and subjects. As you can see, the maximum generally decreases with increasing eccentricity: At the fovea, it is roughly twice the value at 13.5${}^{\circ }$ from the fovea. Thus, we find, as others have (Williams, Artal, Navarro, McMahon, & Brainard, 1996; Navarro, Moreno, & Dorronsoro, 1998), that best image quality is poorer in the peripheral than in the central field. There is also an asymmetry: Image quality is highest about $5^{\circ }$ temporally from the visual axis. This position of highest quality corresponds approximately to the eye's optical axis (Artal, 2014). The higher quality in that part of the visual field is presumably due to lesser astigmatism in the temporal field than in the nasal field due to the optical-visual axis deviation.

For various applications, it is important to know how image quality varies across the visual field when objects are on a smooth surface like a page of text or a display screen. We examined this next. The middle and right panels of Figure 12 show the Strehl Ratio relative to a spherical surface (centered at the eye) and a planar surface (frontoparallel to the visual axis), respectively. We set the distances of those surfaces to best-focus distance (i.e., highest Strehl Ratio) at the fovea. The variation in image quality for different surfaces should be quite dependent on distance to the surface. For example, consider a sphere and plane at distance d from the eye and a ray deviating from the visual axis by angle α. The distance to the spherical surface along the ray is of course d. The distance to the plane is $\frac{d}{cos(\alpha )}$. The difference in those distances in diopters is

(5) $$\Delta D=\frac{1-cos(\alpha )}{d}$$

Thus, as d increases, ΔD decreases, reaching an asymptote at a value of 0 at long distance. This means that differences in image quality for one smooth surface relative to another should diminish with increasing viewing distance. To incorporate this, we examined image quality for three stimulus distances: +4 D (25 cm), +2 D (50 cm), and +1 D (100 cm). Figure 12 shows the results at +2 D, a common distance for text and display screens. Image quality at that distance is lower when the external surface is a sphere or a plane rather than the retinal conjugate surface in the left panel. This means that spherical or planar display screens will produce somewhat poorer image quality across the retina than a display screen whose shape conforms to the conjugate surface (Figure 10). The results for +4 and +1 D are provided in Supplementary Figure S2. As expected, there is a dramatic effect of distance. When the stimulus is near, image quality is substantially better for best-focus distance than for the spherical or planar surface. When the stimulus is far, image quality does not vary substantially. For more on the topic of image quality and planar surfaces, see Thibos (2020).

## Discussion

In this section, we examine the visual consequences of the retinal conjugate surfaces and extend these measured surfaces into the binocular domain introducing the blur horopter. We compare the blur horopter to its analog in binocular vision: the binocular horopter. We investigate whether these horopters conform to the statistics of the natural visual environment. And finally, we use these horopters to refine the concept of the zone of clear single binocular vision.

### Retinal conjugate surface and the visual environment

#### Top-back pitch of retinal conjugate surface and the ground plane

As shown in Figure 10, the retinal conjugate surface is slightly but consistently pitched top-back. Said another way, the emmetropic eye is relatively myopic in the lower visual field and relatively hyperopic in the upper field (Ehsaei et al., 2011; Seidemann et al., 2002). Here we examine whether this is a useful adaptation to a common feature in the natural environment: the ground plane. Given the well-documented ability of visual feedback to modulate the size and shape of the developing eye in many animals (Wallman, Turkel, & Trachtman, 1978; Schaeffel, Glasser, & Howland, 1988), we speculate that the elevation-dependent change in best focus emerges from visual experience with common features such as the ground.

**Figure 13.**
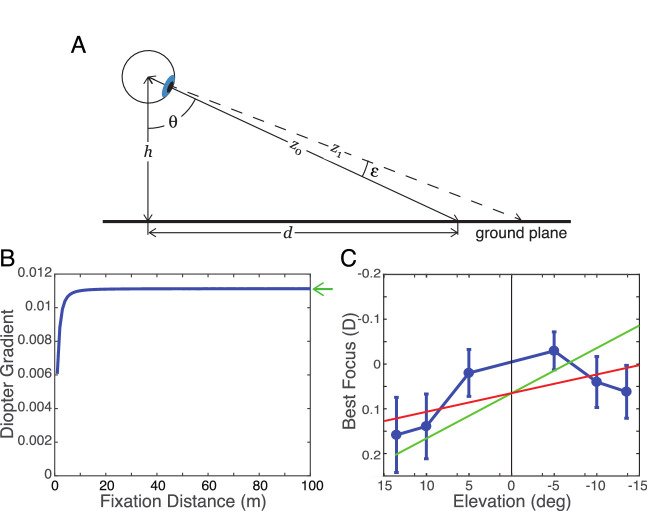
Top-back pitch of the retinal conjugate surface and the ground plane. (**A**) The geometry of the viewing situation. An eye at height h views a point on the ground plane at distance d. The angle between earth vertical and the line of sight is θ. The distance along the line of sight is $z_{0}$. Another visual line is rotated by angle ε from the line of sight. It intersects the ground plane at distance $z_{1}$ from the eye. (**B**) Diopter gradient as a function of fixation distance. The average human eye height h of 1.57 m was used. The eye fixates at different distances d along the ground. The diopter gradient is the change in dioptric distance ($\frac{1}{z_{1}}-\frac{1}{z_{0}}$) per degree of visual angle (ε). The curve asymptotes at a value of 0.011 diopters per degree. The green arrow indicates the value predicted by Equation (9). (**C**) Distance of best focus as a function of elevation. Relative distance of best focus in diopters is the average across six accommodative distances and all emmetropic subjects. The data were calculated from values in three radial directions: vertical and $\pm 30^{\circ }$ from vertical at eccentricities of -13.5,-10,-5,+5,+10, and +13.5${}^{\circ }$. The error bars are standard deviations. The red line is the best-fitting plane to the data. Its slope is 0.0041 diopters per degree. The green line is the diopter gradient associated with the ground plane; its slope is 0.011. An eye height of 4.2 m, more than double actual human eye height, would yield a diopter gradient commensurate with the red line.

In a previous article, we examined whether the ground plane is conjugate to the retinas of ground-dwelling animals (Sprague, Cooper, Reissier, Yellapragada, & Banks, 2016): That is, does the relative myopia and hyperopia of the lower and upper fields render the ground in best focus? The viewing geometry is illustrated in Figure 13A. The eye is at height h above the ground. The line of sight is rotated by θ relative to earth vertical and intersects the ground at distance d from the feet. The distance of that point along the line of sight is $z_{0}$. A visual line slightly higher in the visual field intersects the ground at distance $z_{1}$ from the eye. If the upper visual line is rotated by ε relative to the line of sight:

(6) $$z_{0}=\frac{h}{\cos(\theta )}$$

(7) $$z_{1}=\frac{h}{\cos(\theta +\varepsilon )}$$

Expressing $z_{0}$ and $z_{1}$ in diopters and taking the difference:

(8) $$\Delta D=\frac{\cos(\theta )-\cos(\theta +\varepsilon )}{h}$$

We refer to the change in diopters per degree of elevation as the *diopter gradient*. Figure 13B plots the diopter gradient for the average human eye height of 1.57 m (Buxton, 2015). As you can see, the gradient is nearly constant for all but near distances; to close approximation, it depends only on eye height. Thus, no matter where an upright person looks along the ground (provided that it is not too close), the change in diopters across a vertical patch of the visual field is essentially constant. For large values of d and ε = 1${}^{\circ }$, a useful approximation is

(9) $$\Delta D\approx \frac{\pi }{180h}$$

The green arrow in Figure 13B shows this approximation.

Previous studies reported that the dioptric gradient associated with the ground is consistent with eye shapes in several ground-dwelling animals (Sprague, Cooper, Tošić, & Banks, 2015, 2016). They found the best-fitting line to the spherical equivalent of the refraction as a function of field elevation for several animals. They plotted those elevation-dependent changes in refraction against eye height along with the predictions from Equation (9). There was a striking correspondence between predicted and observed for 1-day, 1-week, 4-week, 6-week, and adult chickens and for adult turtles, guinea pigs, quails, pigeons, horses, and humans.

We now have more accurate measurements of retinal conjugacy in humans for several vertical eccentricities, so we reexamine whether the conjugate surface conforms to the ground plane. Figure 13C plots the relative distance of best focus averaged across six accommodative distances and the 16 subjects. This again shows the relative myopia and hyperopia of the lower and upper visual fields, respectively. The data are concave, but we fit them with a line nonetheless to enable comparison with the prediction for the ground plane. The red line is the fit; its slope is 0.0041 diopters per degree. The asymptotic diopter gradient for the ground plane (Equation (8)) is 0.0113, a value that is larger than, but qualitatively similar to, the gradient of the blur horopter. We conclude that the elevation-dependent change in refraction in humans, like that in many ground-dwelling animals, is somewhat well suited for placing the ground plane in sharp focus as an upright viewer looks from one place on the ground to another. But humans encounter many other common features in the environment, and we will examine this later in the Discussion.

#### Depth of field

The visual system has depth of field: That is, small changes in object distance do not yield discriminable changes in blur even when the eye does not accommodate (Campbell, 1957; Atchison, Charman, & Woods, 1997). Depth of field is larger in the periphery than in the fovea (Wang & Ciuffreda, 2004), so many variations in the distances of objects imaged onto the peripheral retina do not produce noticeably blurred imagery.

We estimated the positions in front of and behind the conjugate surface that are likely to be perceived as not blurred. For this, we used data from studies by Wang and colleagues (Wang & Ciuffreda, 2004, 2005; Wang, Ciuffreda, & Irish, 2006). In these studies, subjects were cyclopleged, which prevented accommodative responses. Stimuli were viewed monocularly through a 5-mm artificial pupil. The focal distance of the fixation target was adjusted to maximize image sharpness, and that distance remained fixed. The peripheral stimulus was a high-contrast circular edge centered on fixation. The edge's radius was varied; those radii defined the retinal eccentricity of the stimulus. To measure thresholds, stimulus distance was increased or decreased until the subject reported that the circular edge appeared blurred. Stimulus size at the retina remained constant as focal distance was manipulated. The just-detectable change in focal distance increased roughly linearly with eccentricity. It is important to note that Wang and Ciuffreda's results are precisely the kind of data required for our purpose. They manipulated the actual focal distance of the stimulus for different retinal eccentricities, so other blurring elements (e.g., astigmatism, higher-order aberrations) were introduced naturally by the viewer's eye and not by rendering them into the stimulus. Thus, their data tell us what natural changes in object distance relative to fixation are detectable.

The defocus thresholds Wang and Ciuffreda reported for the fovea were 0.45–0.85 D, which is much higher than 0.1–0.2 D as reported by many others (Campbell & Westheimer, 1958; Sebastian, Burge, & Geisler, 2015; Walsh & Charman, 1988). Wang and Ciuffreda used an ascending and descending method of limits, a procedure that undoubtedly yielded larger threshold values compared to the forced-choice methods used by the others. To take this methodological difference into account, we multiplied Wang and Ciuffreda's data by 0.14, which produced foveal thresholds consistent with those in the literature.

**Figure 14.**
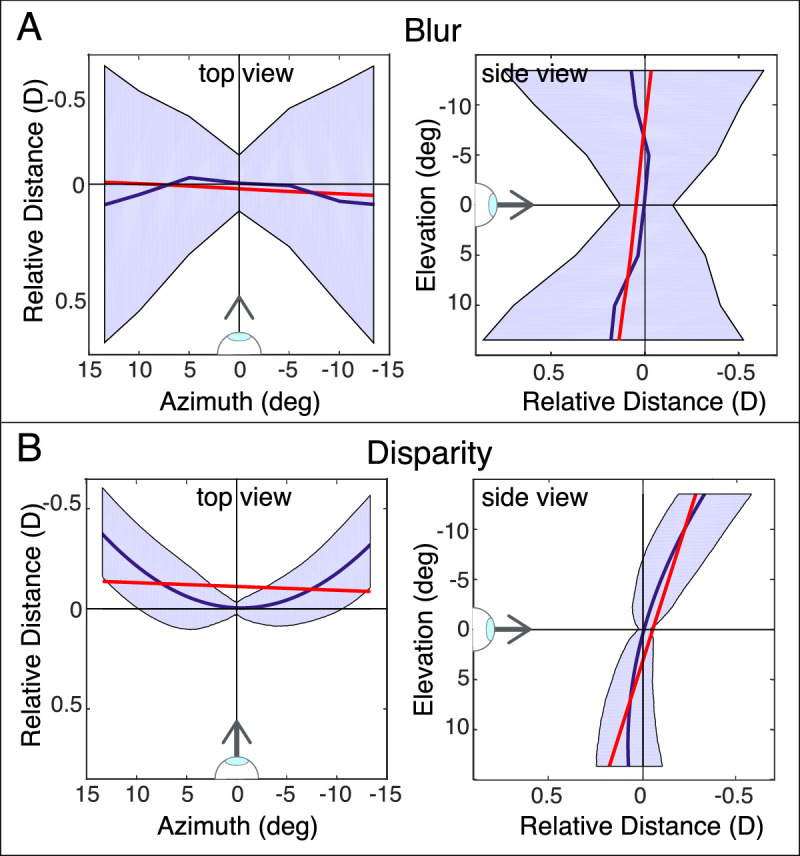
Depth of field and Panum's fusional area. (**A**) Relative best-focus distance for left eye and depth of focus. As in Figure 7, best absolute distance at the fovea has been subtracted from best absolute distance at all other field positions. Data have been averaged across subjects and six accommodative stimuli. The left panel shows the cross section of the best-focus surface along the horizontal meridian for the left eye. The blue curve and blue shaded regions represent the best-focus distances and the range of undetectable changes in the distance. The right panel shows the cross section of the best-focus surface (blue curve) along the vertical meridian and the line fit to those data (red line). Again, the blue shaded region represents the range of stimulus distances that should not yield a detectable change in blur. (**B**) Binocular horopter and Panum's fusion area. The left panel shows a cross section of this horopter (blue curve) along the horizontal meridian and a line fit to the data (red line). The right panel shows the cross section of this horopter (blue) along the vertical meridian and a line fit to those data (red). The data are referenced to the cyclopean eye (the midpoint between the left and right eyes). We fit a second-order polynomial to the horopter data (Gibaldi & Banks, 2019). The equation for the fitted surface is $Z=-0.0020X+0.0160Y-0.0018X^{2}-0.0001XY-0.0009Y^{2}+1000/830$, which yielded $R^{2}$ = 0.927. The blue shaded region is the fusional area: the range of stimulus distances that should not yield diplopia.

Figure 14A shows the results. The dark blue lines in the left and right (top and side views, respectively) panels are cross sections of the best-focus surface—the retinal conjugate surface—at different eccentricities. The shaded zones around those lines are our estimates of the depth of field in front of and behind the best surface. Thickness increases with eccentricity, which means that a considerable range of object distances should be perceived as equally sharp in the peripheral field. The distances in the figure are in diopters relative to best-focus distance, which allows us to plot the expected depth of focus for different accommodative stimuli in the same figure. If we used units of distance (e.g., centimeters), the region of acceptable sharpness would of course be quite large at long stimulus distances.

#### Binocular fusion area

We can perform a similar calculation for binocular disparity. The binocular horopter is the set of positions in the world where rays from corresponding points in the two eyes intersect. Panum's fusion area is the area in front of and behind the horopter where binocular fusion occurs (i.e., images are perceived as single rather than double) (Ogle, 1950). To enable direct comparison of the retinal conjugate surface to the blur horopter, we converted disparity (specifically, horizontal disparity of an object point relative to the fixation point) into diopters (Held et al., 2010).

Figure 14B shows top and side views of the binocular horopter in diopters; the measurements are taken from a previous study (Gibaldi & Banks, 2019). The side view illustrates the well-known top-back pitch of this horopter (Siderov, Harwerth, & Bedell, 1999; Cooper, Burge, & Banks, 2011; Schreiber, Hillis, Filippini, Schor, & Banks, 2008). The top view shows the equally well-known flattening of the horizontal aspect of the binocular horopter relative to the Vieth-Müller Circle (Hillis & Banks, 2001; Schreiber et al., 2008). The shaded areas represent the fusion area where binocular stimuli are seen as single. The measurements in the right panel are from Ogle (1950). We also applied those measurements to represent the fusion area in the left panel. Ogle obtained the fusion data by having the subject binocularly fixate a point straight ahead while adjusting the distance of a rod in the peripheral visual field. The rod was moved closer to the cyclopean eye to find the proximal distance where it first appeared double and was moved farther to find the distal distance where it again appeared double. Those proximal and distal points defined the fusion area for the tested eccentricity. The Ogle data are the type we need because they were obtained by moving real objects forward and backward from the horopter. Thus, natural changes in the retinal images (i.e., greater blur with increasing distance from the conjugate surfaces) were introduced by the viewer's eyes.

The binocular fusion area is much smaller than the depth of field, which means that objects that are moved farther or nearer than the horopter will be perceived as double before they are perceived as blurred (Held, Cooper, & Banks, 2012). This relationship holds for all of the measured field positions.

### Retinal conjugate surface, binocular horopter, and natural scenes

**Figure 15.**
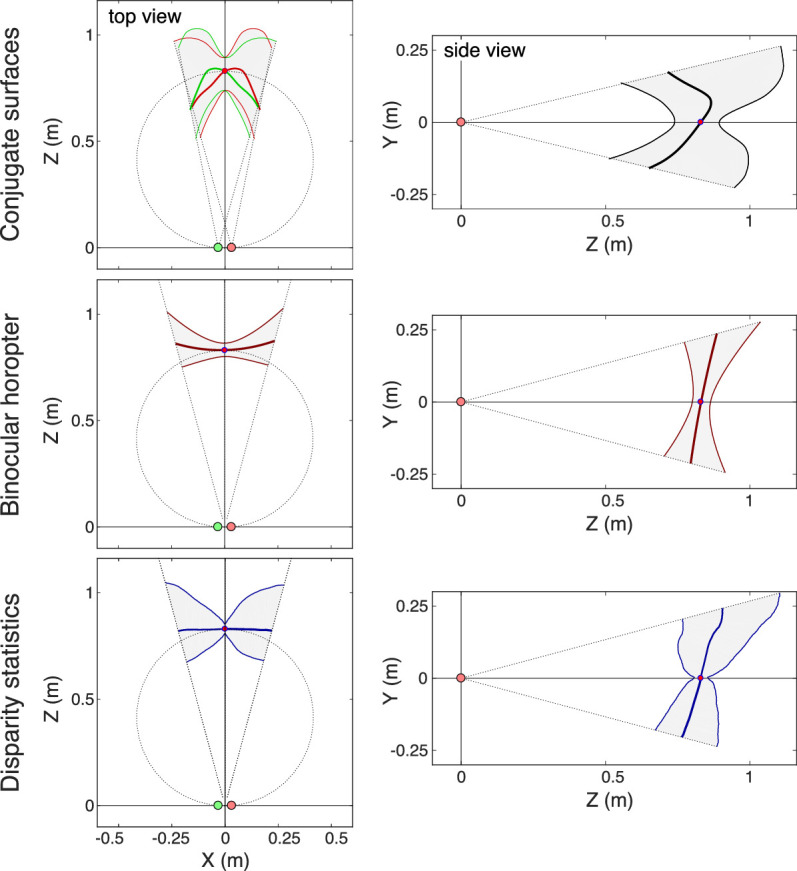
Retinal conjugate surfaces, binocular horopter, and natural-scene statistics. The images were calculated for a field of view of $\pm 13.5^{\circ }$ for the most likely fixation distance (83 cm; 1.20 D) (Sprague et al., 2015; Gibaldi & Banks, 2019) and for an average interpupillary distance of 6.25 cm (Dodgson, 2004). Upper panels: Retinal conjugate surfaces. Top-down view on the left; side view on the right. In the top-down view, the thick green curve represents the surface for the left eye and the thick red curve the one for right eye. The I SAW data are from left eyes only, so the surface for the right eye is simply the mirror image of the one for the left eye. We have subtracted accommodative error at the fovea as in Figure 6. The thin lines represent the near and far limits of the stimulus distances that should yield no detectable change in blur. The circle is the Vieth-Müller Circle. The right panel is a side view. Middle panels: The binocular horopter. The thick curve is the horopter. The thin curves are the near and far limits of the binocular fusion area. We used Ogle's (1950) data directly in the left panel and applied them at equal eccentricities in the right panel. Lower panels: Natural-scene statistics. The thick curves represent median distances from those statistics. The thin curves represent ±1 standard deviation.

Our main objective is to determine where objects in natural viewing are in best focus and the volume around best-focus positions that produce perceptually sharp images. The upper row of Figure 15 shows top and side views of the retinal conjugate surfaces and the depth of field surrounding them in *XYZ* coordinates. They have been plotted for forward gaze at a distance of 0.83 m because those are most likely gaze direction and distance (Sprague et al., 2015; Gibaldi & Banks, 2019). The left and right panels are top and side views, respectively.

The middle row of the figure shows the binocular horopter and Panum's fusion area surrounding it. The left and right panels are again top and side views. The binocular horopter is smoother than the retinal conjugate surfaces, and the volume around it is roughly half the size of the volume around the conjugate surfaces.

We next asked whether those positions in space conform to the distributions of naturally occurring distances in everyday viewing. The bottom row of Figure 15 shows naturally occurring object distances in different parts of the visual field when fixation distance is 0.83 m. The data are from measurements of scene distances and binocular fixations while subjects engaged in four everyday tasks (Gibaldi & Banks, 2019). The thick curve is the median distance for different parts of the visual field. The thin lines in front of and behind the curve are ±1 standard deviation. The median distance and standard deviation of the natural-scene statistics are strikingly similar to the center and volume around the binocular horopter (Sprague et al., 2015; Gibaldi & Banks, 2019). The Root Mean Square error (RMS) between the binocular horopter and the medians of the scene statistics is just 0.092 D/deg${}^{2}$. The retinal conjugate surface and the volume around it are qualitatively but not quantitatively similar to the scene statistics; the conjugate surface is too concave to conform to the statistics. The RMS error between the conjugate surface and the medians of the statistics is 1.733 D/deg${}^{2}$, which is 19 times greater than that for the binocular horopter.

#### The blur horopter

When a sharp image is presented to one eye and a blurred image to the other, the binocular percept is sharp. Specifically, the fused percept is much more similar to the sharp image than to the blurred one (Legge & Rubin, 1981; Legras, Hornain, Monot, & Chateau, 2001; Zheleznyak, Sabesan, Oh, MacRae, & Yoon, 2013). We now incorporate this property of combining the two eyes’ images.

**Figure 16.**
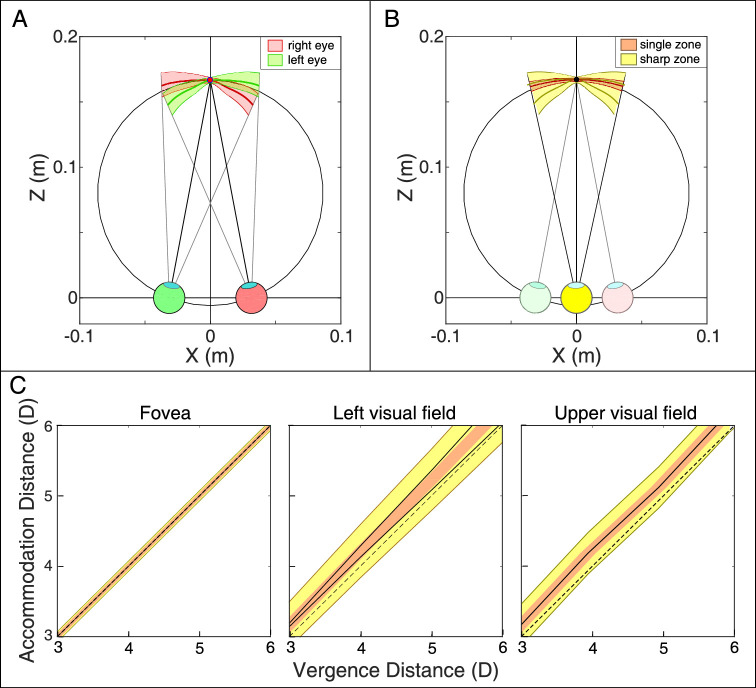
Blur and binocular horopters; zone of clear single binocular vision. (**A**) Retinal conjugate surfaces and depths of field for the two eyes when the eyes are converged at a distance of 6 D (16.7 cm) with an interpupillary distance of 6.25 cm. Field of views are $\pm 13.5^{\circ }$. Thick lines are the conjugate surfaces. Green and red shaded areas represent the depths of field for the left and the right eyes, respectively. The blur horopter is the two retinal conjugate surfaces. An object falling on one of those surfaces will be perceived binocularly as sharp. (**B**) Region of sharp and single vision for vergence at 6 D. The dark yellow lines are the two retinal conjugate surfaces: the blur horopter. The yellow shading around those lines is the region in space for which a binocularly viewed stimulus will be perceived as sharp. The red line is the binocular horopter and the pink area around it the binocular fusion area within which a stimulus will appear single. (**C**) Regions of sharp and single vision in different parts of the visual field. From left to right, at the fovea, 10${}^{\circ }$ to the left of the fovea, and 10${}^{\circ }$ above. The black dashed lines are the natural-viewing lines where the stimuli to vergence and accommodation are the same. The black solid lines represent the two retinal conjugate surfaces, which in combination are the blur horopter. The lines are superimposed in the fovea and upper visual field. They diverge in the left visual field as the stimulus distance increases in diopters (decreases in centimeters). The yellow and pink shaded areas represent the regions in which a stimulus would appear sharp and single, respectively.

The overlapping retinal conjugate surfaces of the two eyes are shown in Figure 16A for a near-fixation distance of 6 D (16.7 cm). The two retinal conjugate surfaces are in combination the *blur horopter*. Objects that fall on one or the other of the two surfaces will be seen as sharp in the binocular percept. Thus, the blur horopter is the position in object space for which objects appear optimally focused to an observer under binocular viewing conditions. At the distance shown in the figure, the distal boundary of the left eye's depth of field extends to greater distance on the right than the boundary of the right eye's field. Also, the distal boundary of the right eye's depth of field extends farther on the left than the boundary of the left eye's. The opposite holds for the proximal boundaries. Because sharp images dominate in the binocular percept, the effective zone of sharpness extends to farther and nearer distances when a viewer looks with two eyes rather than one. Said another way, the zone of sharp vision is extended in binocular viewing. Where and by how much the extension with binocular viewing occurs depends on fixation distance. Figure 16B shows the regions that should be seen as both sharp and single.

The *zone of clear single binocular vision* (*ZCSBV*) is the range of vergence and accommodative stimuli for which the viewer has sharp, single vision (Hofstetter, 1945; Peli, 1995; Gifford, Gifford, Hendicott, & Schmid, 2020). Outside of the zone, the percept is blurred and/or diplopic. In graphs of the ZCSBV, distance to the vergence stimulus is plotted in diopters on the horizontal axis and distance to the accommodative stimulus on the vertical axis. A typical ZCSBV has a width of about 2 D (Hofstetter, 1945). It contains motor and sensory components. The motor component is the degree to which the viewer can make vergence and accommodative responses to conflicting distances, which may require undoing vergence-accommodation coupling (Schor, 1992). The sensory component represents the tolerance between stimulus and response before the percept becomes blurred and/or diplopic. For example, the binocular fusion zone represents the range of disparities that can be tolerated before the percept becomes diplopic. We can use our data and analysis to accurately quantify the sensory component of the ZCSBV for different parts of the visual field. Results are shown in Figure 16C. The left, middle, and right panels represent the sensory component of the ZCSBV for the fovea, 10${}^{\circ }$ to the left of the fovea, and 10${}^{\circ }$ above the fovea. The pink regions represent the range of disparities (plotted as vergence distances) that would appear single (i.e., binocularly fused) for each vergence and accommodative stimulus distance. The thickness is determined horizontally. The yellow regions represent the range of focal distances (plotted as accommodative distances) that would appear sharp for each vergence and accommodative stimulus distance. The black lines represent the retinal conjugate surfaces for the two eyes, which in combination is the blur horopter. They diverge at near distance (large diopter values), which means that the region of sharpest vision is expanded in binocular viewing because when an object falls on one conjugate surface and not the other, it will be seen binocularly as sharp. The thickness of the yellow region is determined vertically. As you can see, the regions are larger in the peripheral field positions than in the fovea, and the regions of sharp vision are larger than that of single vision. The foveal zone we calculated is much smaller than the typical ZCSBV (Hofstetter, 1945) because ours concerns natural viewing where vergence and accommodative responses are quite accurate (Labhishetty et al., 2020), whereas the typical zone is measured by clinicians who insert conflicts between vergence and accommodation by placing prisms or lenses in front of the viewer's eyes, thereby measuring the combined contributions of motor and sensory components.

#### How often are images seen as blurred or double?

**Figure 17.**
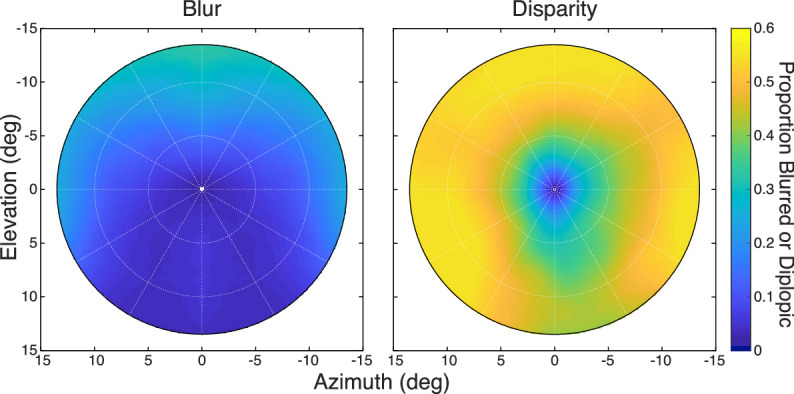
Proportion of naturally occurring stimuli seen as blurred or diplopic. Left: The proportion of stimuli from the natural-scene statistics that should be seen as blurred given the shape of the blur horopter and the depth of field. The scene statistics are the weighted combination across four natural tasks (Gibaldi & Banks, 2019). To make this figure, we fit a second-order polynomial to the horopter data. The equation for the surface is $Z=0.0062X-0.0064Y+0.0009X^{2}+0.0001XY+0.0008Y^{2}+1000/830$, where Z is distance in diopters, and X and Y are azimuth and elevation in degrees, which yielded $R^{2}$ = 0.955 between the data and surface. The depth of field measures are from previous studies (Wang & Ciuffreda, 2004, 2005; Wang et al., 2006). Right: The proportion of stimuli from the natural-scene statistics that should be seen as double given the shape of the binocular horopter and Panum's fusion area. The binocular horopter was represented by the second-order polynomial fitted to data from Gibaldi and Banks (2019) for a fixation distance of 83 cm. The limits for Panum's fusion area are from Figure 14.

We next examined how likely it is to perceive blurred or diplopic images in natural viewing. We used the blur and binocular horopters to define surfaces of best focus and binocular correspondence, respectively. We expressed the distances of points on those surfaces in diopters relative to fixation distance, which enables us to combine data across fixation distances and to directly compare blur and binocular data. We then applied the depth of field, the binocular combination rule (“sharp wins”), and the binocular fusion area of Figure 14B to estimate volumes relative to the horopter surfaces. Finally, we used the distributions of relative distances at each field position from natural-scene statistics (Gibaldi & Banks, 2019) to determine the proportion of naturally occurring distances that should be seen as blurred or double. The results are shown in Figure 17. The probability of perceiving blur in natural viewing is quite low: ∼0.1 within 5${}^{\circ }$ of the fovea and ∼0.2 within 10–13${}^{\circ }$ of the fovea. The probability is lower in binocular than in monocular viewing because the binocular combination rule serves to broaden the range of sharp vision. The probability of perceiving double images is greater: ∼0.2 within 2${}^{\circ }$ of the fovea climbing to ∼0.5 within 10–13${}^{\circ }$ of the fovea. The probability of seeing blurred or double imagery is lower in the lower than the upper visual field. This occurs because the variance of naturally occurring distances in the lower field is less than the variance in the upper field (Figure 15) (Sprague et al., 2015; Gibaldi & Banks, 2019). Thus, most naturally occurring scenes are perceived as sharp and single, particularly in the central visual field.

## Conclusion

We used measurements of optical aberrations across the central visual field of emmetropic eyes to define the retinal conjugate surface: the positions in space where objects create the highest image quality. Expressed in diopters, the shape of the region does not change substantially as the eye accommodates from near to far. It is pitched top-back such that best focus is farther in the upper field than in the lower. It is also rotated temporal side back such that best focus is farther in the temporal field than in the nasal.

We introduced the binocular extension of the retinal conjugate surface: the blur horopter. We examined the degree to which the blur horopter and binocular horopter conform to the distribution of naturally occurring distances. We found, as others have (Sprague et al., 2015; Gibaldi, Canessa, & Sabatini, 2017; Gibaldi & Banks, 2019), close correspondence between the binocular horopter and natural-scene statistics. We found qualitative but not quantitative agreement between the blur horopter and the statistics. We offer two explanations for the differing degrees of correspondence. First, the volume of acceptable sharpness around the blur horopter is much larger than the volume of binocular fusion around the binocular horopter (Figures 14 and 15). This means, of course, that many more naturally occurring distances are seen as sharp than are seen as fused (Figure 17). As a consequence, the visual feedback provided by the environment is stronger for the binocular horopter than for the blur horopter. Second, binocular correspondence is determined by connections among binocular neurons in visual cortex, and these can be readily modified in development by pruning early proliferate connections (Cowan, Fawcett, O'Leary, & Stanfield, 1984). In contrast, modification of retinal conjugate surfaces requires changes in the shape of the retina and/or changes in the physical structure of the crystalline lens and cornea. These changes are subject to other constraints (i.e., maintaining a roughly spherical eye shape to enable eye rotations and to maintain adhesion of the retina to posterior structures). Thus, the way change is implemented may be more readily accomplished in the binocular horopter than in the blur horopter, making the former more amenable to visual experience.

## Supplementary Material

Supplement 1

Supplement 2
