# Supplementary material for: The blur horopter: Retinal conjugate surface in binocular viewing
Source: J Vis. 2021 Mar 4;21(3):8. doi: 10.1167/jov.21.3.8 (PMC7938023; doi:10.1167/jov.21.3.8)
Supplement: Supplement 2 [file jovi-21-3-8_s002.pdf]

Journal of Vision (2021) 21(3):8, 1-21

The Blur Horopter: Retinal Conjugate Surface in Binocular Viewing

Agostino Gibaldi School of Optometry, University of California at Berkeley, Berkeley, CA, USA

Vivek Labhishetty School of Optometry, University of California at Berkeley, Berkeley, CA, USA

Larry N. Thibos School of Optometry, Indiana University, Bloomington, IN, USA, USA

Martin S. Banks School of Optometry, Vision Science Program, University of California at Berkeley, Berkeley, CA, USA

Supplementary Materials

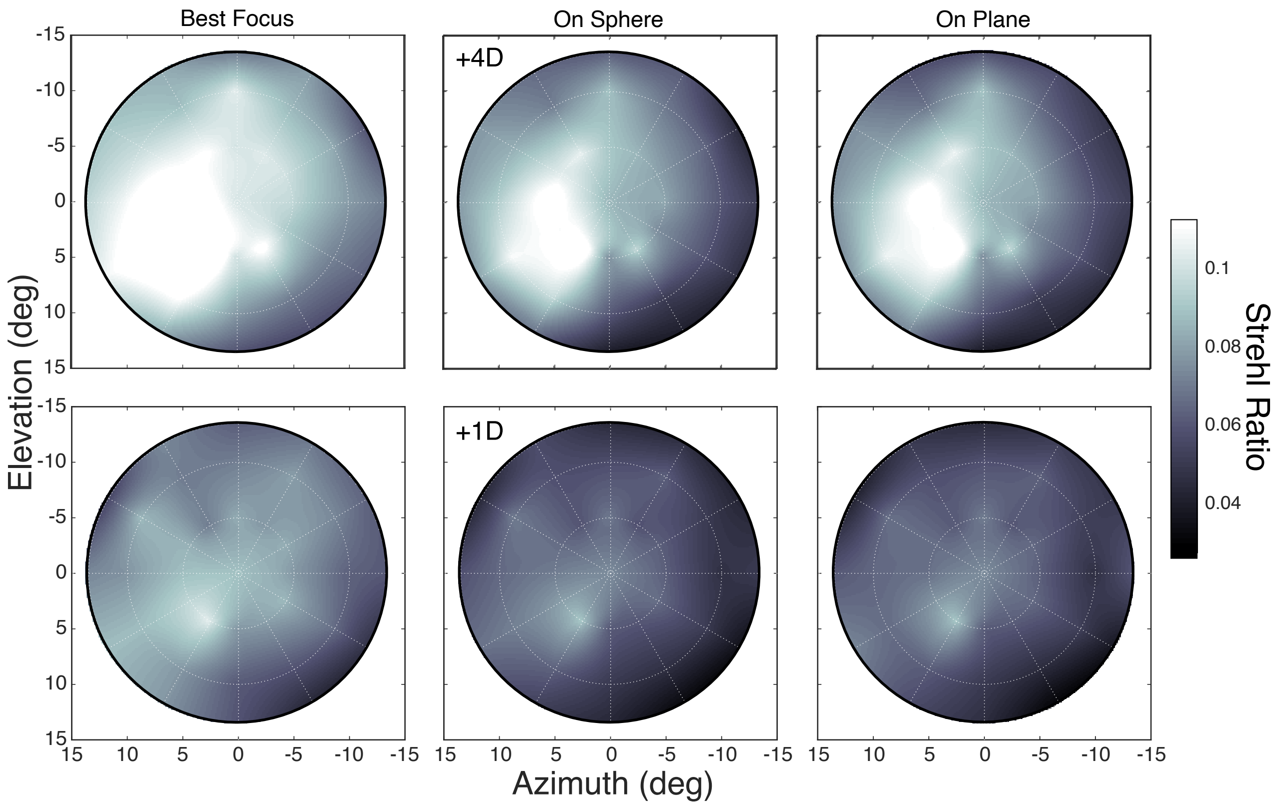

Figure S1: Image quality across the visual field for accommodative stimulus distances of +4D and +1D. Each panel shows the Strehl Ratio for all field positions averaged across subjects. Lighter values correspond to higher Ratios and therefore better image quality. The upper row shows the Ratios for a stimulus at +4D (25cm) and the lower row the Ratios for +1D (100cm). From left to right, the panels show the Strehl Ratios at best-focus distance, the Ratios relative to a spherical surface (with the fovea in best focus), and the Ratios relative to a planar surface (again with the fovea in best focus). The spherical and planar reference surfaces are the same as those in Fig. 2C,D. U, N, L, and T indicate upper, nasal, lower, and temporal visual field, respectively.

https://doi.org/10.1167/jov.21.3.8 ISSN 1534–7362 Copyright 2021 The Authors
